# Interfacial electronic states and self-formed asymmetric Schottky contacts in polar α-In_2_Se_3_/Au contacts

**DOI:** 10.1038/s41598-023-46514-0

**Published:** 2023-11-06

**Authors:** Sha Han, Cai-Juan Xia, Min Li, Xu-Mei Zhao, Guo-Qing Zhang, Lian-Bi Li, Yao-Heng Su, Qing-Long Fang

**Affiliations:** 1 School of Science, Xi’an Polytechnic University, Xi’an, 710048 Shaanxi China; 2 Engineering Research Center of Flexible Radiation Protection Technology, University of Shaanxi Province, Xi’an Polytechnic University, Xi’an, 710048 Shaanxi China; 3 Xi’an Key Laboratory of Nuclear Protection Textile Equipment Technology, Xi’an Polytechnic University, Xi’an, 710048 Shaanxi China

**Keywords:** Nanoscale materials, Theory and computation

## Abstract

In recent years, the two-dimensional (2D) semiconductor α-In_2_Se_3_ has great potential for applications in the fields of electronics and optoelectronics due to its spontaneous iron electrolysis properties. Through ab initio electronic structure calculations and quantum transport simulations, the interface properties and transport properties of α-In_2_Se_3_/Au contacts with different polarization directions are studied, and a two-dimensional α-In_2_Se_3_ asymmetric metal contact design is proposed. When α-In_2_Se_3_ is polarized upward, it forms an n-type Schottky contact with Au. While when α-In_2_Se_3_ is polarized downward, it forms a p-type Schottky contact with Au. More importantly, significant rectification effect is found in the asymmetric Au/α-In_2_Se_3_/Au field-effect transistor. The carrier transports under positive and negative bias voltages are found to be dominated by thermionic excitation and tunneling, respectively. These findings provide guidance for the further design of 2D α-In_2_Se_3_-based transistors.

## Introduction

Ferroelectric materials, which exhibit the spontaneous and reversible polarization, have great potential in a wide range of applications, such as non-volatile memory^1,2^, field-effect transistors^3^, and optoelectronic devices^4,5^. With the increasingly urgent need for device miniaturization, it is necessary to further explore ultrathin or nanoscale ferroelectric materials. However, the conventional perovskite ferroelectric materials, such as Pb(Zr,Ti)O_3_^6^, BaTiO_3_^7^, and BiFeO_3_^8^, suffer from the size effect. Due to the depolarization field arising from an incomplete screening of surface charges, the out-of-plane polarization of the material will disappear when the film thickness is less than a few nanometers. This becomes a major obstacle for the scaling of ferroelectric-based devices.

In recent years, In_2_Se_3_ has attracted much attention as an emerging two-dimensional (2D) III–VI ferroelectric semiconductor material. Ding et al.^9^ predicted that the single-layer In_2_Se_3_ exhibits room-temperature ferroelectricity with reversible spontaneous electric polarization in both out-of-plane and in-plane orientations. Distinct from other 2D and conventional bulk ferroelectric materials, In_2_Se_3_ exhibits intrinsically inter-correlated in-plane and out-of-plane polarizations, and the reversion of the out-of-plane polarization by a vertical electric field also causes the rotation of the in-plane polarization^10^. Due to the non-centrosymmetry originating from the hexagonal stacking, the co-existence of in-plane and out-of-plane piezoelectricity also occurs in α-In_2_Se_3_ ultrathin crystals^11^. Wan et al.^12^ observed the Schottky barrier at the graphene/α-In_2_Se_3_ interface that can be effectively tuned by switching the electric polarization under an applied voltage. 2D In_2_Se_3_-based field effect transistors (FETs) have been demonstrated with high ON/OFF ratio of 10^8^ and on-state current of 671 μA·μm^−1^^13^. Poh et al.^14^ designed an asymmetric ferroresistive memory device to exploit the 2D semiconductor ferroelectric properties of α-In_2_Se_3_ and achieved outstanding performances, with a giant electroresistance ratio of 3.9 × 10^6^ and a readout current density of > 12 A/cm^2^, which enables the convenient fabrication of high-performance ferroelectric Schottky diode memory applications.

In semiconductor devices, metal–semiconductor (MS) junctions/interfaces play a pivotal role^15,16^. One strategy produces asymmetric current via metal–semiconductor-metal (MSM) structures, which consists of two MS junctions with distinct Schottky barriers connected back to back^17,18^. One Schottky barrier MS interface in the MSM structure is forward biased when a non-zero bias voltage is applied, while the other one is reversed biased. Predictably, the asymmetric MSM structure can be achieved when polar materials are contacted with the same metal.

In this paper, the interface properties and electron transport properties of α-In_2_Se_3_/Au contacts with different polarization directions are systematically investigated. Our results show that the α-In_2_Se_3_/Au contact can be switched from n-type to p-type Schottky contact when α-In_2_Se_3_ polarization direction is reversed from upward to downward. Moreover, our transport calculations show that the intrinsic dipole results in asymmetric I–V characteristics, further underscoring the asymmetry of the MSM structure. The results provide helpful insights in the future design and fabrication of α-In_2_Se_3_-based new-concept devices.

## Computational method and model

The calculations are performed using the projector-augmented wave (PAW)^19^ pseudopotential and the plane wave cutoff energy of 400 eV as implemented in the Vienna ab initio simulation package (VASP)^20,21^. The Perdew-Burke-Ernzerhof (PBE) formulation of the generalized gradient approximation (GGA) is used to describe the exchange–correlation interaction^22^. The Brillouin-zone integration is performed by using the 5 × 5 × 1 Monkhorst–Pack k-mesh and a Gaussian smearing broadening of 0.05 eV is adopted. The crystal lattice are relaxed to the ground state using a conjugate gradient algorithm. The total energy is converged to less than 1.0 × 10^−5^ eV and the maximum force is less than 0.02 eV/Å during optimization. A 15 Å vacuum layer is adopted in the z direction to avoid interaction between periodic slabs. The long-range van der Waals (vdW) forces in α-In_2_Se_3_/Au contacts are described by the DFT-D2 method of Grimme^23^.

Transport simulations are performed using the QuantumATK 2015 package^24^ to perform the density functional theory (DFT) coupled with non-equilibrium Green’s function (NEGF) method^25,26^. The two-probe model is applicable to study the Schottky barrier in the transmission system in a FET. Here, a 5 nm freestanding single-layer α-In_2_Se_3_ is set as the channel (both up/down directions of single-layer α-In_2_Se_3_ are taken into considerations) and α-In_2_Se_3_/Au interfacial system is considered as the electrode^27^. The periodic, Neumann, and Dirichlet boundary conditions are used in the x, y, and z directions of the device, respectively. In x direction, the single layer α-In_2_Se_3_ is selected and a 15 Å vacuum layer is adopted to avoid interaction between periodic slabs. The lattice constant is 7.630 Å in y direction and the gate length of 66.075 Å is selected in z direction. The transmission coefficient $T^{k} (E)$ is obtained from the Green's function as follows:

1 $$ T^{k} \left( E \right) = {\text{Tr}}\left[ {\Gamma_{L}^{k} (E)G^{k} \left( E \right)\Gamma_{R}^{k} (E)G^{k} {}^{\dag }\left( E \right)} \right] $$

where *k* is set as a the reciprocal-lattice vector point along a surface-parallel direction which is also orthogonal to the transmission direction in the irreducible Brillouin zone (IBZ). *G*^*k*^(*E*) and *G*^*k* †^(*E*) represent the retarded and advanced Green’s functions, respectively. Based on the self-energies $\sum {_{L/R}^{k} }$, $\Gamma_{{L{/}R}}^{k} (E) = {\text{i}}(\Sigma_{{L{/}R}}^{r,k} - \Sigma_{{L{/}R}}^{a,k} )$ describes the spectral function of the left and the right electrodes, respectively^28^. Single-ξ polarized (SZP) basis set is employed for high accuracy. The electronic structures of the electrodes and central region are calculated with a Monkhorst–Pack 50 × 50 × 1 and 1 × 50 × 1 k-point grids, respectively. The real-space mesh cutoff is set to 150 Ry.

**Figure 1:**
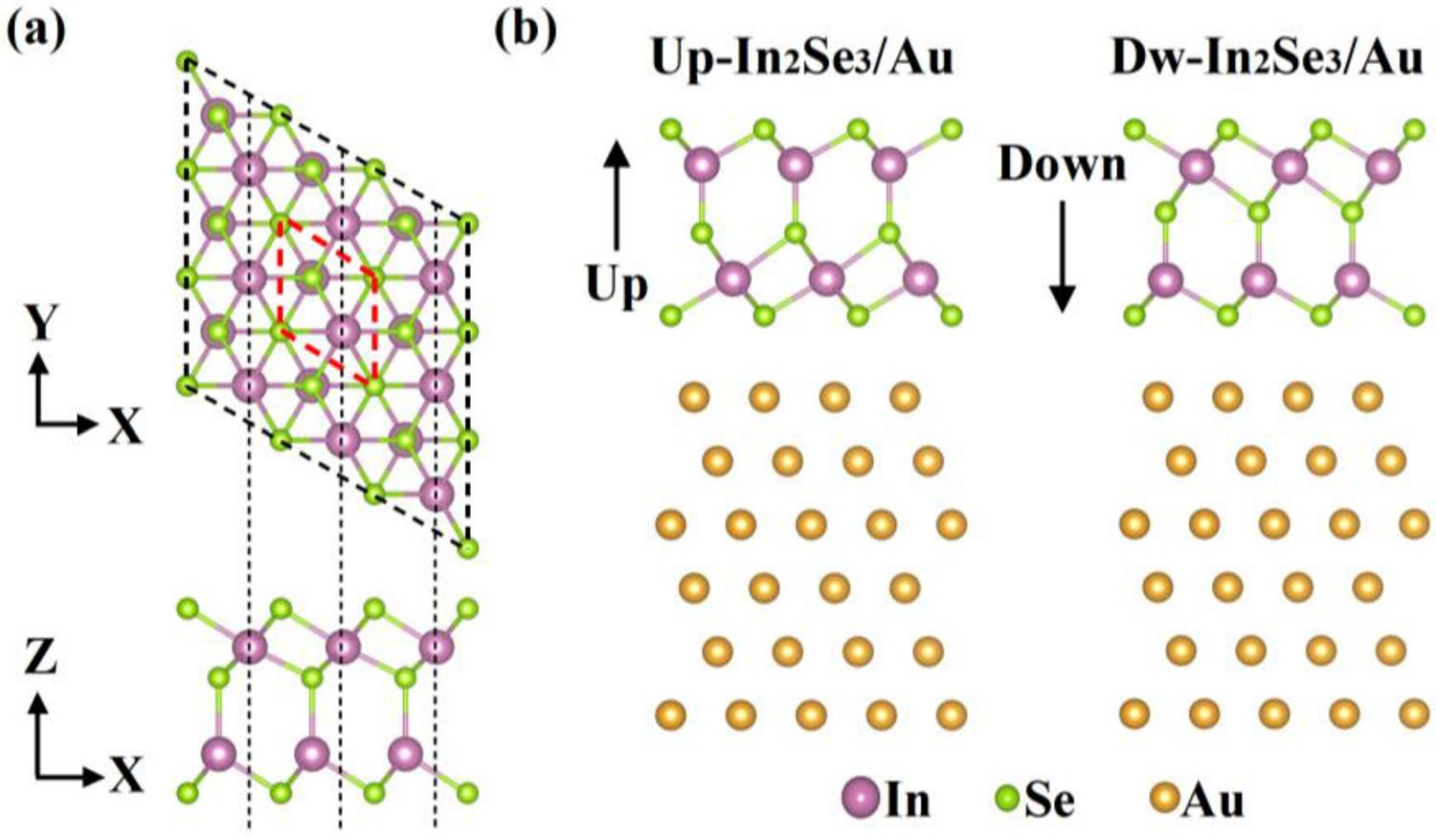
(**a**) Top and side views of the crystal structure of α-In_2_Se_3_. (**b**) Side views of α-In_2_Se_3_/Au contacts with two different ferroelectric polarization directions.

In the single-layer α-In_2_Se_3_, the five atomic sublayers are arranged in sequence of Se–In–Se–In–Se in the out-of-plane direction, while atoms are arranged in a triangular lattice in each atomic sublayer, as shown in Fig. 1a. The optimized lattice constant of single-layer α-In_2_Se_3_ is a = 3.986 Å, which is consistent with previous studies^29^. The polarization direction of α-In_2_Se_3_ is defined by the direction of the built-in electric field. We construct the α-In_2_Se_3_/Au contact by considering two different ferroelectric polarization directions, as shown in Fig. 1b, denoted as Up-In_2_Se_3_/Au and Dw-In_2_Se_3_/Au. To limit the lattice mismatch between α-In_2_Se_3_ and Au, the supercell matching patterns are set as follows: The 4 × 4 unit cell of Au (111) face is adjusted to the 3 × 3 unit cell of α-In_2_Se_3_ with a lattice mismatch of less than 2.610%.

## Results and discussion

The α-In_2_Se_3_/Au structure has been fully optimized to obtain the energetically favorable configuration. During the process, the strain due to the lattice mismatch between the two layers is released by a small adjustment of the atomic configuration. The equilibrium interlayer distance (*d*_z_) is the average distance between the closest layer of α-In_2_Se_3_ and Au in the vertical direction. *d*_Se-M_ is set as the minimum distance between the selenium atoms in α-In_2_Se_3_ layer and the atoms in the Au layer. The binding energy ($E_{{\text{b}}}$) of the α-In_2_Se_3_/Au contact is defined as:

2 $$ E_{{\text{b}}} = (E_{{{\text{In2Se3}}}} + E_{{{\text{Au}}}} - E_{{{\text{In}}_{{2}} {\text{Se}}_{{3}} {\text{/Au}}}} )/N $$

where $E_{{{\text{In}}_{{2}} {\text{Se}}_{{3}} }}$, $E_{{{\text{Au}}}}$ and $E_{{{\text{In}}_{{2}} {\text{Se}}_{{3}} /{\text{Au}}}}$ represent the total energies of the α-In_2_Se_3_ layer, Au layer, and α-In_2_Se_3_/Au heterostructure, respectively. The total number of unit cells of α-In_2_Se_3_ in the system is represented by *N*. According to this definition, positive values denote favorable interface binding. As shown in Table 1, the *d*_z_ and *d*_Se-M_ are 2.523 Å (2.665 Å) and 2.643 Å (2.697 Å) for Up-In_2_Se_3_/Au (Dw-In_2_Se_3_/Au) contact, respectively, and they are comparable with the covalent bond length of Au-Se (2.56 Å). While for the isolated Au (5*d*^10^6*s*^1^) atoms, the outermost orbital is occupied by only one unpaired *s*, which leads to a relatively strong *E*_b_ because only one covalent bond will be formed between Au and α-In_2_Se_3_. Furthermore, the *E*_b_ are 0.962 eV and 0.822 eV for Up-In_2_Se_3_ and Dw-In_2_Se_3_ contacted with Au, respectively.

**Table 1:** Calculated interfacial properties of α-In_2_Se_3_ on Au surfaces.

System	Up-In_2_Se_3_/Au	Dw-In_2_Se_3_/Au
Mismatch (%)	2.610	2.610
*d*_z_ (Å)	2.523	2.665
	2.760^a^	2.920^a^
	2.275^b^	2.485^b^
*d*_Se-M_ (Å)	2.643	2.697
	3.340^a^	3.400^a^
*E*_b_ (eV)	0.962	0.822
	0.510^a^	0.350^a^
Δ*ρ* (10^–3^ e/Å^2^)	4.455	3.518
*W*_Au_ (eV)	5.152	5.152
*W*_Se-Au_ (eV)	4.598	5.344
$\Phi_{{{\text{L}} ,{\text{T}}}}^{{\text{e}}}$(eV)	0.524	0.723
$\Phi_{{{\text{L}} ,{\text{T}}}}^{{\text{h}}}$(eV)	0.724	0.445
*E*_g_ (eV)	1.248	1.168

The lattice constant mismatches are given. The interlayer distance *d*_z_ is the averaged distance between the surface Se atoms of α-In_2_Se_3_ and the lowest Au layer in the z direction. The minimum distance *d*_Se-M_ is the distance between the topmost Se atom of α-In_2_Se_3_ and lowest Au atom. *E*_b_ is the binding energy per unit cell of α-In_2_Se_3_. Δ*ρ* is the charge transfers from Au layer to α-In_2_Se_3_ layer in Up- and Dw-In_2_Se_3_/Au contacts. *W*_Au_ (eV) and *W*_Se-Au_ (eV) are the work functions of Au and the α-In_2_Se_3_/Au systems. $\Phi_{{{\text{L}} ,{\text{T}}}}^{{\text{e}}}$(eV) and $\Phi_{{{\text{L}} ,{\text{T}}}}^{{\text{h}}}$(eV) are the lateral electron (hole) Schottky barrier height (SBH) obtained from the quantum transport simulation. *E*_g_ (eV) is the transport band gap of the FET.

^a^DFT value from ref.^27^.

^b^DFT value from ref.^30^.

**Figure 2:**
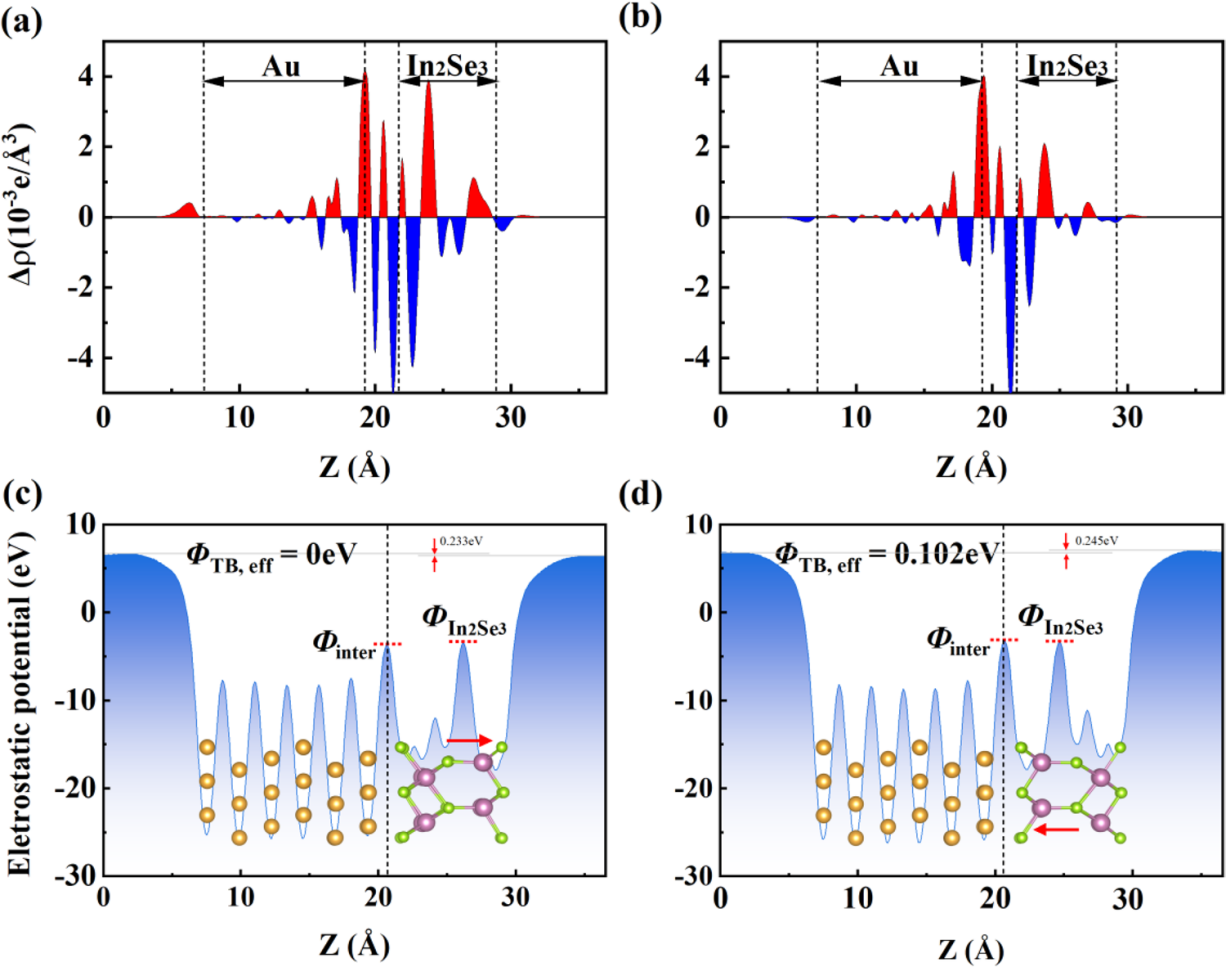
Electronic structure at the interface between α-In_2_Se_3_ and Au. ∆*ρ* is the planar average charge density difference between α-In_2_Se_3_ and Au. *V*_eff_ is the electrostatic potential along the z-direction for α-In_2_Se_3_ contacted with Au. (**a**,**c**) for Up-In_2_Se_3_/Au contact, as well as (**b**,**d**) for Dw-In_2_Se_3_/Au contact.

To gain further insight into interfacial properties, the plane-averaged charge density difference is calculated as follows:

3 $$ {\Delta }\rho = \rho_{{{\text{In}}_{{2}} {\text{Se}}_{{3}} {\text{/Au}}}} - \rho_{{{\text{Au}}}} - \rho_{{{\text{In}}_{{2}} {\text{Se}}_{{3}} }} $$

where $\rho_{{{\text{In}}_{{2}} {\text{Se}}_{{3}} {\text{/Au}}}}$, $\rho_{{{\text{In}}_{{2}} {\text{Se}}_{{3}} }}$, and $\rho_{{{\text{Au}}}}$ are the charge densities of the α-In_2_Se_3_/Au contact, single-layer α-In_2_Se_3_, and Au layer, respectively. Figure 2a,b show the charge density difference of Up-In_2_Se_3_/Au and Dw-In_2_Se_3_/Au contacts, respectively. Apparently, charge transfer occurs near the interface of α-In_2_Se_3_ contacted with Au, indicating the formation of interfacial dipole. Bader charge analysis shows that the charge transfer in Up-In_2_Se_3_/Au contact (4.455 × 10^−3^ e/Å^2^) is larger than that in Dw-In_2_Se_3_/Au contact (3.518 × 10^−3^ e/Å^2^), as listed in Table 1. The result is in accordance with the interlayer distance and binding energy.

The tunneling barrier is crucial to the electrical properties of the semiconductor/metal contact. The tunneling barrier height, $\Phi_{{\text{TB, eff}}}$, is defined as the lowest barrier to be overcome by the electrons from Au to the α-In_2_Se_3_, calculated as the potential difference between interface ($\Phi_{{{\text{inter}}}}$) and the potential energy of α-In_2_Se_3_ ($\Phi_{{{\text{In}}_{{2}} {\text{Se}}_{{3}} }}$)^30,31^. The $\Phi_{{{\text{In}}_{{2}} {\text{Se}}_{{3}} }}$ is higher than $\Phi_{{{\text{inter}}}}$ in Up-In_2_Se_3_/Au contact, indicating no tunneling barrier exists, as shown in Fig. 2c. However, $\Phi_{{\text{TB, eff}}}$ of the Dw-In_2_Se_3_/Au contact is 0.102 eV, as show in Fig. 2d.

**Figure 3:**
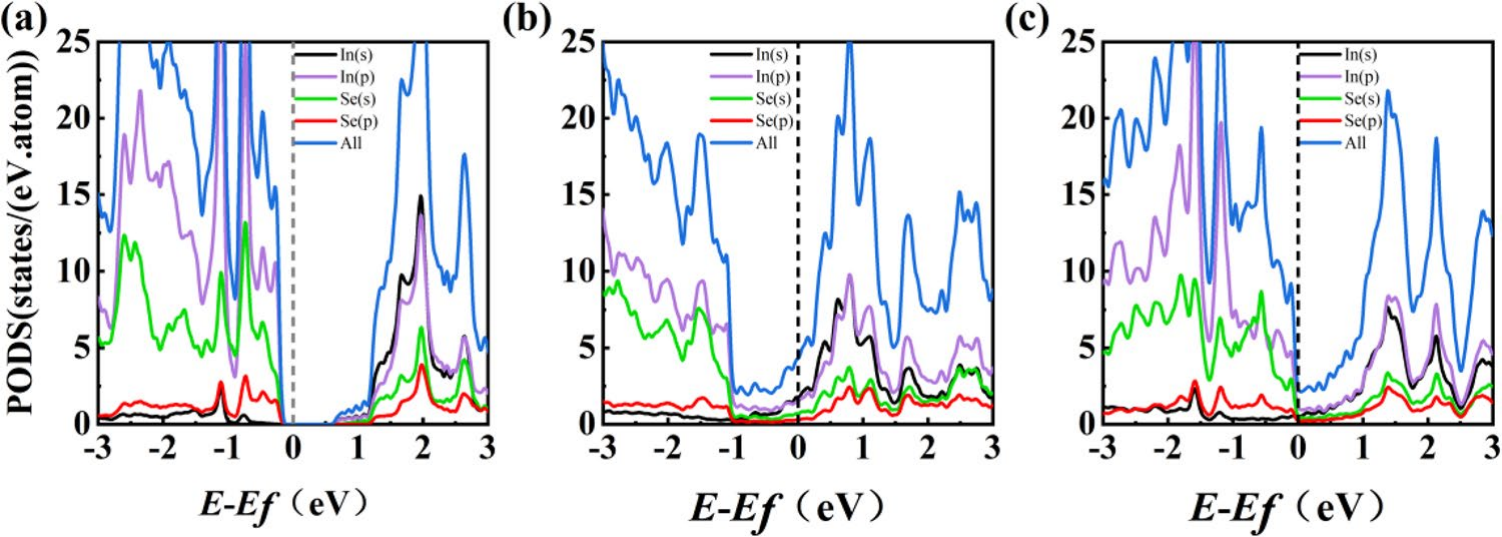
Projected density of states (PDOS) of α-In_2_Se_3_ contacted with Au. (**b**) Up-In_2_Se_3_/Au and (**c**) Dw-In_2_Se_3_/Au. The Fermi level *E*_F_ is set at 0 eV and indicated by the black vertical dashed line. (**a**) The PDOS of freestanding monolayer α-In_2_Se_3_ is provided for comparison.

**Figure 4:**
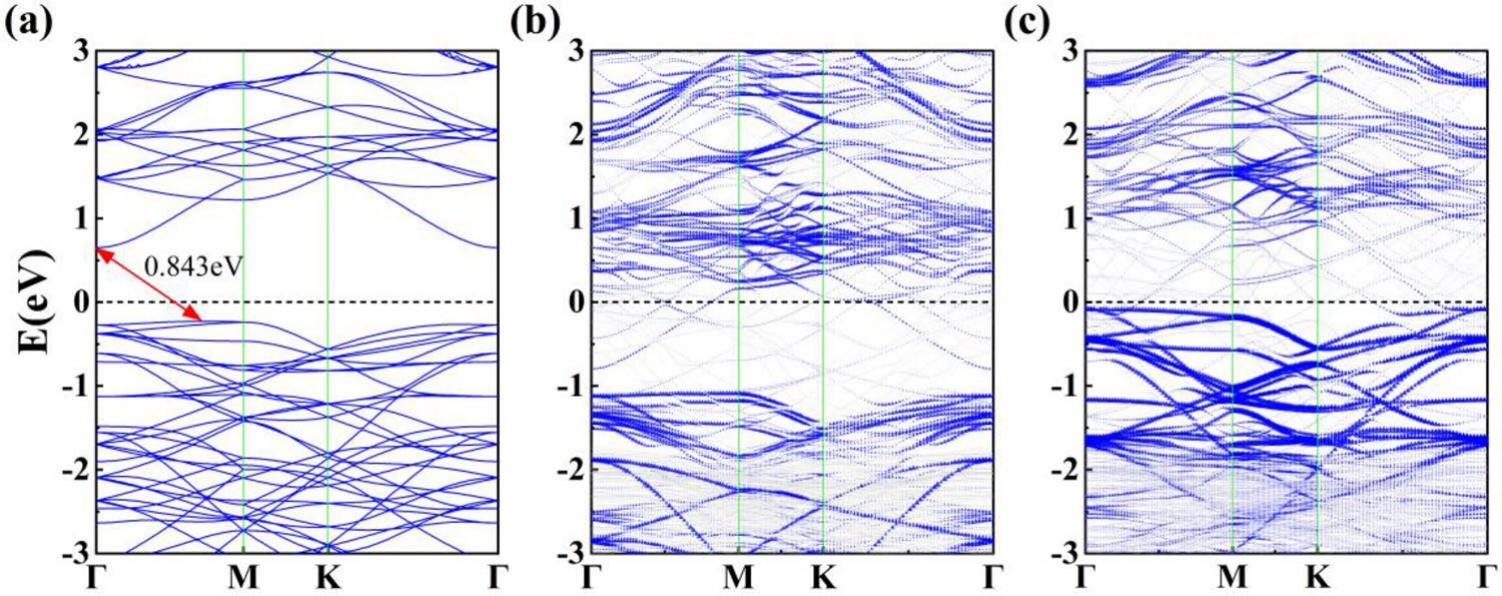
Band structure of of α-In_2_Se_3_ contacted with Au. (**b**) Up-In_2_Se_3_/Au and (**c**) Dw-In_2_Se_3_/Au. The Fermi level is set to zero energy. The blue line indicates the energy band structure of the α-In_2_Se_3_ projection, whose weight is represented by the size of the dot. (**a**) The band structure of freestanding monolayer α-In_2_Se_3_ is provided for comparison.

Figure 3 shows the projected density of states (PDOS) of the freestanding single-layer α-In_2_Se_3_ and Up- and Dw-In_2_Se_3_/Au contacts. The conduction band portion of bulk α-In_2_Se_3_ is mainly contributed by In *s* and In *p*, while the valence band portion is mainly contributed by In* p* and Se *s.* Compared with the independent α-In_2_Se_3_, the *E*_F_ of the Up-In_2_Se_3_/Au heterostructure can be found to be shifted in the direction of the conduction band, but the *E*_F_ of the Dw-In_2_Se_3_/Au heterostructure is shifted toward the valence band. The indirect band gap of the freestanding monolayer α-In_2_Se_3_ is 0.843 eV, and the valence band maximum (VBM) of α-In_2_Se_3_ is located between the Γ and M points in the first Brillouin zone, while the conduction band minimum (CBM) is located at the Γ point, which is consistent with previous theoretical results^9^, as shown in Fig. 4a. Figure 4 shows the energy band structures of (b) Up-In_2_Se_3_/Au and (c) Dw-In_2_Se_3_/Au heterostructures. The blue and gray colors represent the contributions from the α-In_2_Se_3_ and Au layers, respectively. Compared with pristine α-In_2_Se_3_, the energy bands of α-In_2_Se_3_ hybridize with Au metal to some extent, but most of the α-In_2_Se_3_ energy bands and Au energy bands can be identified by different colors. It can be found that the Up-In_2_Se_3_ energy band structure is significantly more hybridized than Dw-In_2_Se_3_, which is consistent with the strong interfacial forces analyzed previously. The energy band structure of α-In_2_Se_3_ is all disrupted and the Fermi energy level crosses the α-In_2_Se_3_-dominated energy band, indicating that α-In_2_Se_3_ undergoes metallization in all these interfacial systems.

**Figure 5:**
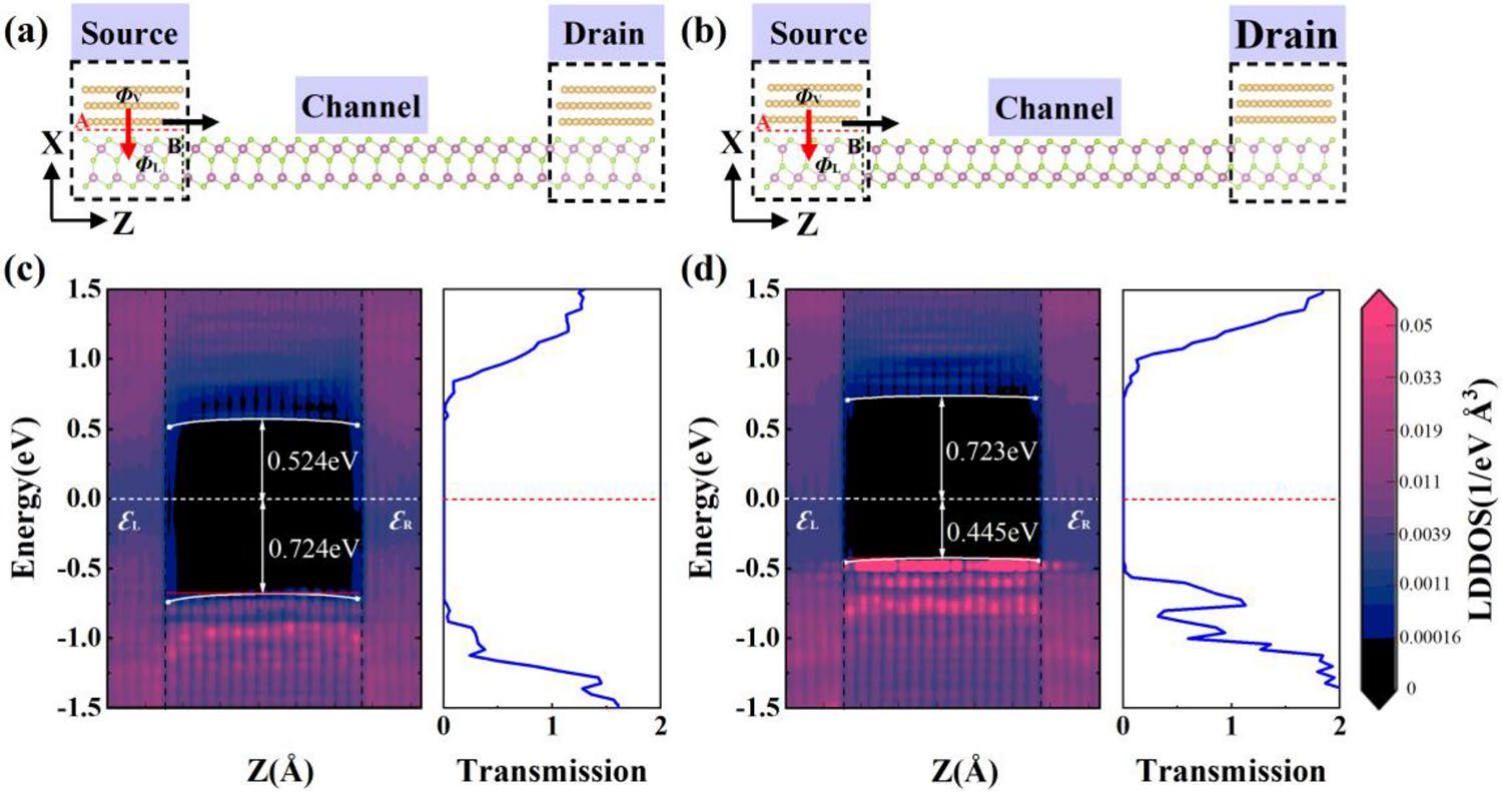
Transport properties of α-In_2_Se_3_/Au field-effect transistor with a channel length of 5 nm. Schematic cross-sectional view of (**a**) Up-In_2_Se_3_/Au and (**b**) Dw-In_2_Se_3_/Au. The red and black arrows show the pathway of electron injection from Au to α-In_2_Se_3_ at interfaces A and B, respectively. $\Phi_{{\text{V}}}$ and $\Phi_{{\text{L}}}$ represent the SBH in the vertical and lateral direction, respectively. Local device density of states (LDDOS) and transmission spectra of (**c**) Up-In_2_Se_3_/Au and (d) Dw-In_2_Se_3_/Au under zero bias.

In the above electronic structure calculation, the electronic properties of the electrode region and channel are treated separately, ignoring the coupling between the two regions. A more reliable approach to study transistor SBH is to use the DFT-coupled NEGF method within a two-probe model, where the composite electrode (α-In_2_Se_3_/Au heterostructure) and the channel semiconductor are calculated as a whole, taking into account the coupling between the electrode region and the channel. As shown in Fig. 5a,b, the two-probe models of Up- and Dw-In_2_Se_3_ FETs are constructed with symmetric MSM structure, respectively. Charge injected from Au electrodes to the channel α-In_2_Se_3_ come across two interfaces (A and B): interface A is between the Au and α-In_2_Se_3_ layer under the metal electrodes; interface B is between the α-In_2_Se_3_ layer under the metal electrodes and the channel α-In_2_Se_3_ layer. α-In_2_Se_3_ undergoes metallization owing to a relative strong interaction with Au electrode, so there is no Schottky barrier $\Phi_{{\text{V}}}$ in the vertical direction and only Schottky barrier $\Phi_{{\text{L}}}$ in the lateral direction.

In general, there are two methods to calculate the transverse SBH. One is the work function approximation (WFA) and the other is the quantum transport simulation (QTS). In WFA, the electron (hole) SBH is simply calculated as the difference between the Fermi level value of the composite electrode and the CBM (VBM) value of the α-In_2_Se_3_ channel. A transverse n-type Schottky contact is formed between Up-In_2_Se_3_/Au with an electron SBH of -0.530 eV, and a transverse p-type Schottky contact is formed between Dw-In_2_Se_3_/Au with a hole SBH of 0.393 eV. In WFA, the electrode-channel coupling is neglected. In contrast, the QTS method takes into account the electrode-channel coupling, so the method is a more reliable way to calculate the Schottky barrier.

Localized device density of states (LDDOS) is a direct method of reflecting the real space energy-band distribution in FETs, and the LDDOS of Up- and Dw-In_2_Se_3_ transistors under zero gate and drain bias are shown in Fig. 5c,d, respectively. The lateral electron SBH ($\Phi_{{{\text{L}},{\text{T}}}}^{{\text{e}}}$) of α-In_2_Se_3_ transistors are described by the difference between the CBM and the Fermi level *E*_F_ of the lateral interface B. Meanwhile, the lateral hole SBH ($\Phi_{{{\text{L}},{\text{T}}}}^{{\text{h}}}$) of α-In_2_Se_3_ transistors are determined by the difference between the VBM and the Fermi level *E*_F_. An n-type Schottky contact is formed at the interface B in Up-In_2_Se_3_ transistor with $\Phi_{{{\text{L}},{\text{T}}}}^{{\text{e}}}$ of 0.524 eV. While the p-type Schottky contact is formed at the interface B in Dw-In_2_Se_3_ transistor with $\Phi_{{{\text{L}},{\text{T}}}}^{{\text{h}}}$ of 0.445 eV. The lateral SBH and polarity from LDDOS calculations are consistent with those extracted from the transmission spectrum.

**Figure 6:**
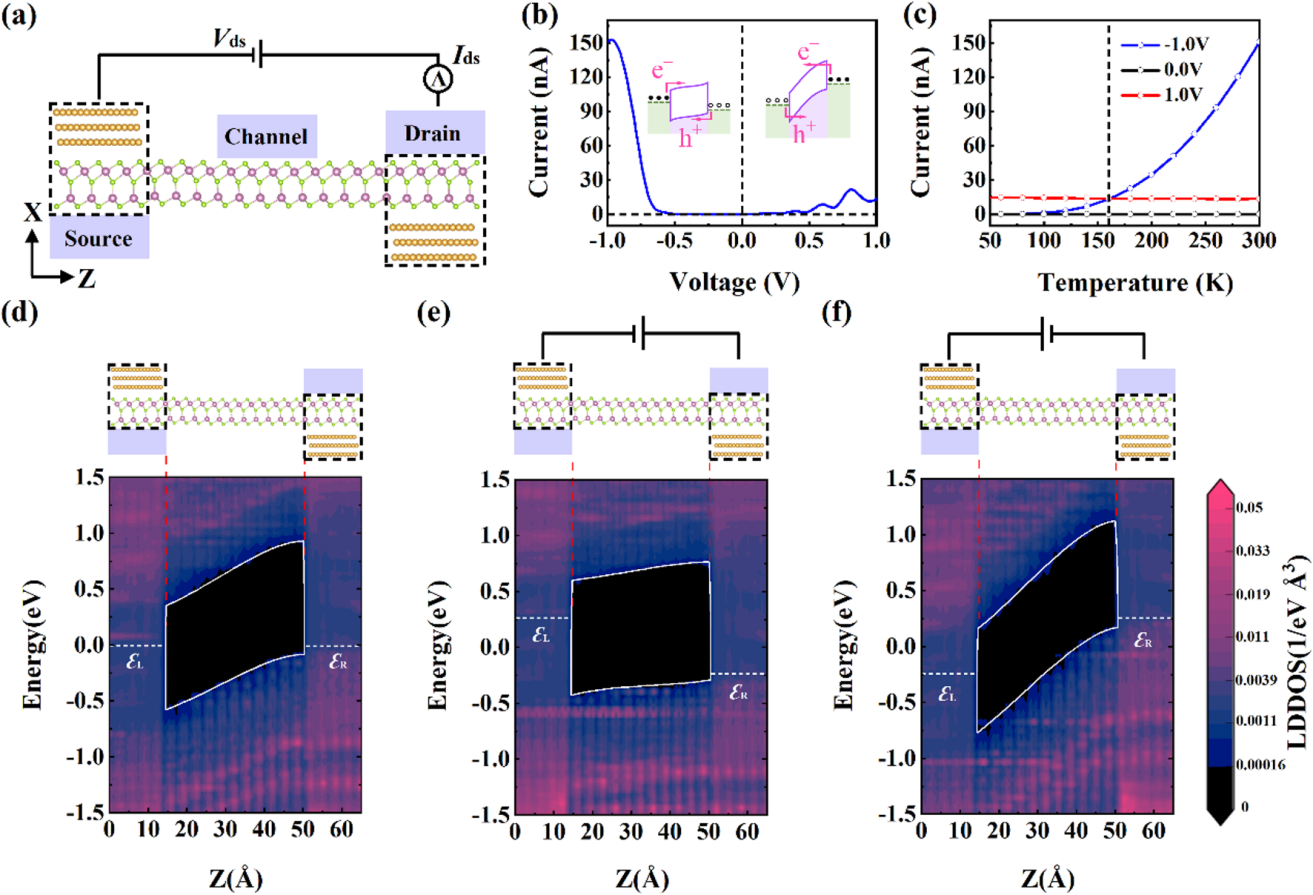
Transport properties of the asymmetric Au/α-In_2_Se_3_/Au FET and transport mechanism. (**a**) Scheme of the asymmetric Au/α-In_2_Se_3_/Au FET. (**b**) The corresponding I–V characteristic curve of this device. The illustrations are schematic band diagrams under negative (left) and positive (right) bias voltages, respectively. (**c**) The current versus temperature from 50 to 300 K of this device under different bias voltages. (**d**–**f**) LDDOS of this device at the bias voltage 0 V, − 1 V and 1 V, respectively.

Figure 6a shows Au/α-In_2_Se_3_/Au FET with asymmetric MSM structure, which has two contacts: Up-In_2_Se_3_/Au (left) and Dw-In_2_Se_3_/Au (right). Since Up-In_2_Se_3_/Au and Dw-In_2_Se_3_/Au exhibit different SBH, this structure yields asymmetric I–V characteristic. Figure 6b shows that the backward current *I*_ds_ (150 nA under − 1 V) is one order of magnitude larger than the forward current *I*_ds_ (10 nA under 1 V). Therefore, the Au/α-In_2_Se_3_/Au FET with asymmetric structure exhibits remarkable rectification performance. At negative voltage, electrons drift from source to drain, crossing the n-type barrier of Up-In_2_Se_3_/Au, while holes drift in the opposite direction and cross the p-type barrier of Dw-In_2_Se_3_/Au. At a positive voltage, the potential barriers for electrons and holes increase. Figure 6c shows that under positive bias, the *I*_ds_ varies slightly with increasing temperature. Under negative bias, the *I*_ds_ has a much greater sensitivity to temperature. The results indicate a different transport mechanism between forward and reverse carrier transport in the MSM structure. To discern the transport mechanisms contributing to the current under each condition, Fig. 6d–f plot LDDOS at bias voltages 0 V and ± 1 V, respectively. It can be seen that the conduction and valence bands at the metal–semiconductor interface tend flat [Fig. 1e and left illustration in Fig. 1b], indicating that the carrier transport under negative bias (− 1 V) is mainly through thermionic excitation^32^. On the contrary, the conduction and valence bands near the interface bend downward with the barrier becoming sharp under positive bias (1 V) [Fig. 1f and right illustration in Fig. 1b], indicating that the carrier transport under positive voltage is mainly induced by tunneling^33^.

## Conclusions

In conclusion, we investigate the interface and transport properties of the Up- and Dw-In_2_Se_3_/Au contacts based on ab initio calculations and quantum transport simulations. α-In_2_Se_3_ has an intrinsic dipole due to its asymmetric structure and can act as either an n-type or p-type diode depending on the stacking structure (Up-In_2_Se_3_/Au vs. Dw-In_2_Se_3_/Au). Our transmission simulations show that a transverse n-type Schottky contact $\Phi_{{{\text{L}},{\text{T}}}}^{{\text{e}}}$ = 0.524 eV is formed in the Up-In_2_Se_3_/Au FET, while a transverse p-type Schottky contact $\Phi_{{{\text{L}},{\text{T}}}}^{{\text{h}}}$ = 0.445 eV is formed in the Dw-In_2_Se_3_/Au FET. The asymmetric I-V characteristics in the asymmetric Au/α-In_2_Se_3_/Au FET exhibits remarkable rectification performance, and transports with hot ion excitation and tunneling under negative and positive bias, respectively. Our study demonstrates a simple and practical method to introduce asymmetric Schottky barriers with the MSM structure, and proposes a conceptual framework, which can be extended to other 2D polar semiconductors.

## Data Availability

The datasets used and/or analysed during the current study available from the corresponding author on reasonable request.
